# Health Risk Assessment of Inhalation Exposure to Formaldehyde and Benzene in Newly Remodeled Buildings, Beijing

**DOI:** 10.1371/journal.pone.0079553

**Published:** 2013-11-14

**Authors:** Lihui Huang, Jinhan Mo, Jan Sundell, Zhihua Fan, Yinping Zhang

**Affiliations:** 1 Institute of Built Environment, Department of Building Science, Tsinghua University, Beijing, China; 2 Key Laboratory of Eco Planning & Green Building, Ministry of Education (Tsinghua University), Beijing, China; 3 Built Environmental Test Center, Tsinghua University, Beijing, China; 4 Department of Environmental and Occupational Medicine, Robert Wood Johnson Medical School, Environmental and Occupational Health Sciences Institute, Rutgers University, Piscataway, New Jersey, United States of America; Indian Institute of Toxicology Reserach, India

## Abstract

**Objective:**

To assess health risks associated with inhalation exposure to formaldehyde and benzene mainly emitted from building and decoration materials in newly remodeled indoor spaces in Beijing.

**Methods:**

We tested the formaldehyde and benzene concentrations in indoor air of 410 dwellings and 451 offices remodeled within the past year, in which the occupants had health concerns about indoor air quality. To assess non-carcinogenic health risks, we compared the data to the health guidelines in China and USA, respectively. To assess carcinogenic health risks, we first modeled indoor personal exposure to formaldehyde and benzene using the concentration data, and then estimated the associated cancer risks by multiplying the indoor personal exposure by the Inhalation Unit Risk values (IURs) provided by the U.S. EPA Integrated Risk Information System (U.S. EPA IRIS) and the California Office of Environmental Health Hazard Assessment (OEHHA), respectively.

**Results:**

(1) The indoor formaldehyde concentrations of 85% dwellings and 67% offices were above the acute Reference Exposure Level (REL) recommended by the OEHHA and the concentrations of all tested buildings were above the chronic REL recommended by the OEHHA; (2) The indoor benzene concentrations of 12% dwellings and 32% offices exceeded the reference concentration (RfC) recommended by the U.S. EPA IRIS; (3) The median cancer risks from indoor exposure to formaldehyde and benzene were 1,150 and 106 per million (based on U.S. EPA IRIS IURs), 531 and 394 per million (based on OEHHA IURs).

**Conclusions:**

In the tested buildings, formaldehyde exposure may pose acute and chronic non-carcinogenic health risks to the occupants, whereas benzene exposure may pose chronic non-carcinogenic risks to the occupants. Exposure to both compounds is associated with significant carcinogenic risks. Improvement in ventilation, establishment of volatile organic compounds (VOCs) emission labeling systems for decorating and refurbishing materials are recommended to reduce indoor VOCs exposure.

## Introduction

Indoor air quality (IAQ) is important for public health because most people spend over 80% of lifetime indoors [1]–[4]. Carbonyls and BTX (benzene, toluene and xylene), a subset of volatile organic compounds (VOCs), represent an important group of indoor air pollutants [5]–[8]. The emission sources of these compounds in indoor environment include building materials, decoration and renovation materials (e.g., vinyl floor and composite wood boards, adhesives, synthesized resins, paints, carpets, furniture) and consumer products (e.g., freshly dry cleaned clothes, mothball and deodorizers) [1], [2], [9]–[17]. Indoor carbonyls can also be formed via ozonolysis of alkenes and terpenes [1], [14], [15], [18], [19]. Inhalation exposure to these compounds may result in a variety of acute and chronic adverse health effects [9], [12], [20], [21] such as Sick Building Syndrome (SBS) symptoms [2], [13], mucous membrane and lower respiratory irritation [1], [12], neurologic effects [2], [12], allergic effects [2], [12], [13], developmental and reproductive effects [12] as well as potential carcinogenic effects (e.g., lung cancer and childhood leukemia) [1], [2], [12], [13].

During the economic boom in the past decades, China has experienced the largest industrialization and urbanization ever in human history [22]. The rapid economic growth and the dramatically increased household wealth result in a nationwide real estate boom. For example, more than 10 million square meters of newly built residential properties were sold each year in Beijing since 2000 (Beijing Statistical Yearbook 2000–2010). Accompanied with the real estate boom is a high demand for building decoration, renovation and refurbishment [23]. Therefore, the IAQ of newly remodeled buildings has become a major public concern in the cities in China. Exposure to formaldehyde in newly remodeled dwellings is suspected to be one of the main causes for the increased childhood leukemia incidence in Chinese mega-cities in recent years [12], [13]. The unhealthy IAQ may also be one of the major causes for a 56% increase of lung cancer incidence in Beijing from 2000 to 2010 [24]. This dramatic increase cannot be fully explained by either ambient VOCs pollution or smoking. In fact, ambient concentrations of VOCs and carbonyls in Beijing have decreased in recent years due to implementation of vehicle emission regulation policy [25]–[27]; the smoking rate for Chinese adults has not significantly changed in the past 15 years [28]. It is challenging to evaluate the acute and chronic health risks from indoor inhalation exposure to VOCs in mega-cities such as Beijing, because it is lack of IAQ monitoring data. In particular, there are few studies on the health risk assessment of IAQ in newly remodeled buildings in China.

The objective of this study was to assess the health risks from inhalation exposure to formaldehyde and benzene in newly remodeled homes and offices in Beijing. The risk assessment was conducted based on the IAQ test results in this study. The reasons to select formaldehyde and benzene as the target VOCs are because they are the main indoor VOC pollutants regulated by GB/T 18883-2002 (the *Chinese National Indoor Air Quality Standard*) [29], and have been ranked top on the list of VOCs with cancer risk potency greater than 1 per million population in the U.S. [10], [20], [21].

## Methods

### Buildings Sampled

**Figure 1:**
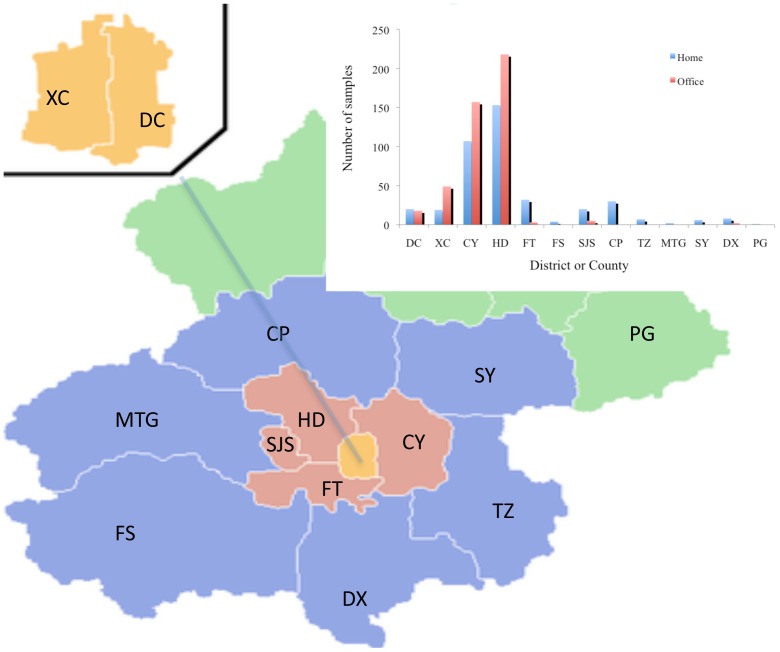
Distribution of the sampled buildings in 13 districts and counties of Beijing. Abbreviation in the figure: XC: Xicheng District, DC: Dongcheng District, HD: Haidian District, CY: Chaoyang District, FT: Fengtai District, FS: Fangshan District, SJS: Shijingshan District, CP: Changping District, TZ: Tongzhou District, MTG: Mengtougou District, SY: Shunyi District, DX: Daxing District, PG: Pinggu County.

The formaldehyde and benzene concentrations in indoor air of 410 dwellings and 451 offices in Beijing were tested from July 2008 through September 2012 by Built Environment Test Center, Tsinghua University. The tested dwellings and offices were remodeled (i.e., renovated, decorated and/or refurbished) within the past year. The IAQ tests were requested by the occupants, who had health concerns on the IAQ. The specific reasons they requested such a test were not asked by the study team but likely included the following: uncomfortable odor, awareness of emissions of VOCs in newly remodeled buildings even without obvious odor, and general concern about the impact of VOC emissions on health, particularly families with vulnerable residents (e.g. infants, kids and elder people). These buildings were located in 13 different districts in Beijing (Figure 1), with 73% dwellings and 98% offices in Chaoyang, Haidian, Dongcheng and Xicheng Districts that are urban areas of Beijing. The field tests were conducted based upon permissions from the property owners, and authorized by Certification and Accreditation Administration of the People’s Republic of China (CMA) and China National Accreditation Service for Conformity Assessment (CNAS).

All tested homes utilized natural ventilation. Split type air conditioners were used for cooling in the summer, except very limited luxury serviced apartments that used central air-conditioning. All homes utilized central heating in the winter. Central air-conditioning was utilized in about 65% of the tested office buildings, while about 35% of the tested office buildings utilized split type air conditioners. It is noted that windows are usually closed in the winter when central heating is used and in the summer when air conditioner is on. Thus, the air exchange rates (AERs) of dwellings in Beijing in the two seasons are expected to be lower than in spring and fall. Different from dwellings, most of the tested offices utilized central air conditioning, and these office rooms were in a closed environment. Therefore, the AERs of these “closed” offices are expected to be low as well. However, we acknowledge that the effects of ventilation on the indoor VOCs concentration cannot be evaluated in this study, since little data on AERs of the dwellings and offices in China is currently available.

### Sample Collection and Chemical Analysis

Field tests were designed and implemented according to the *Chinese National Indoor Air Quality Standard* (GB/T 18883-2002). Prior to the tests, the occupants were asked to stop smoking, remove consumer products that could release VOCs (e.g. mothballs, cleaning products and air fresher). The impacts of human activities on IAQ were minimized as much as possible until completion of the tests. In addition, the occupants were asked to close doors and windows and turn off air conditioning (if they could) for 12–24 hours prior to the IAQ test. Therefore, the indoor environments were in an airtight state during the entire IAQ tests.

Collection of the air samples for formaldehyde and benzene analysis were conducted based on the *Chinese National Indoor Air Quality Standard* (GB/T 18883-2002). The formaldehyde air samples were analyzed using the *Methods for determination of formaldehyde in air of public places* (GB/T 18204.26-2000); whereas the benzene air samples were analyzed based on the *Ambient air-Determination of benzene and its analogies using sorbent adsorption thermal desorption and gas chromatography* (HJ 583-2010). Briefly, duplicate samples were collected for 45 minutes with a sampling rate of 200 mL/minute from the bedrooms in the dwellings. Additional rooms, such as living rooms, were sampled based on the size of the dwellings. Compounds were collected for 45 minutes with a sampling rate of 200 mL/minute from one to five locations in the tested offices, depending on the office size. Samplers were placed at about 1 m above the floor, located as centrally as possible given logistic constraints. Benzene was collected onto Tenax-TA sorbent bed and analyzed using thermodesorption-GC/MS (Series 6850; Agilent Technologies). Formaldehyde was absorbed by 3-methyl-2-benzothiazolinonehydrazone hydrochloride (MBTH) solution and analyzed using UV-VIS spectrometry at 630 nm. Details of the analysis methods are described elsewhere [29]–[31].

### Data Analysis

The measurements obtained from multiple rooms in the dwellings were averaged for both formaldehyde and benzene. If multiple offices in one building were tested, they were regarded as different microenvironments with different occupants. Descriptive statistical analysis was performed for the concentrations of both species. Student T test was conducted to compare the formaldehyde and benzene concentrations between the dwellings and the offices. Since the data were not normally distributed, the lognormal transformed formaldehyde and benzene concentrations were used for analysis. All statistical analysis was conducted by SAS v9.0 (SAS Corporation, Cary, NC), and thereafter.

### Health Risk Assessment

#### Non-carcinogenic health risk assessment

The non-carcinogenic health risks associated with IAQ of the tested buildings were evaluated in terms of the threshold mechanisms of toxic effects [32]. Quantitative risk characterization involves a simple calculation of a hazard index (HI) [32]

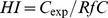
(1)where C_exp_ represents inhalation exposure level of a given air toxic species and RfC represents a “threshold dose” of a given air toxic species. When HI is less than one, it may be inferred that such an exposure is unlikely at risk of toxicity or a given health problem, and vice versa [32].

We compared the indoor concentrations of formaldehyde and benzene to the reference concentrations (RfCs) defined by GB/T 18883-2002 (Chinese National Indoor Air Quality Standard), the RfCs for chronic inhalation exposure defined in the U.S. EPA Integrated Risk Information System (IRIS; U.S. EPA 2010), and the reference exposure levels (RELs) suggested by the California Office of Environmental Health Hazard Assessment (OEHHA; California Environmental Protection Agency, 2008). These reference values are summarized in the Supporting Information (Table S1).

#### Cancer risk assessment

Cancer risks posed by inhalation exposure to formaldehyde and benzene originate from the very exposure in both outdoor and indoor microenvironments, which include, but not limited to, ambient, home, office and transportation. In our study, we focused on cancer risks associated with indoor (i.e. home+office) exposure. For the assessment of cancer risks from indoor exposure to formaldehyde and benzene, it is necessary to obtain personal concentrations of the two compounds in indoor microenvironments. Personal concentrations can be determined by two methods. The first method is personal exposure measurement, which was not feasible in this study due to many factors such as available human subjects and willingness of participation. The second method is to a) use computation modeling to simulate human’s daily activity and air pollution in microenvironments; and b) derive the distribution of personal concentrations of the two compounds from the distributions of human activity and air toxics concentrations in indoor microenvironments [21], [33]. Monte Carlo is a frequently used simulation method, as it can generate numerical distribution through repeated random sampling.

**Figure 2:**
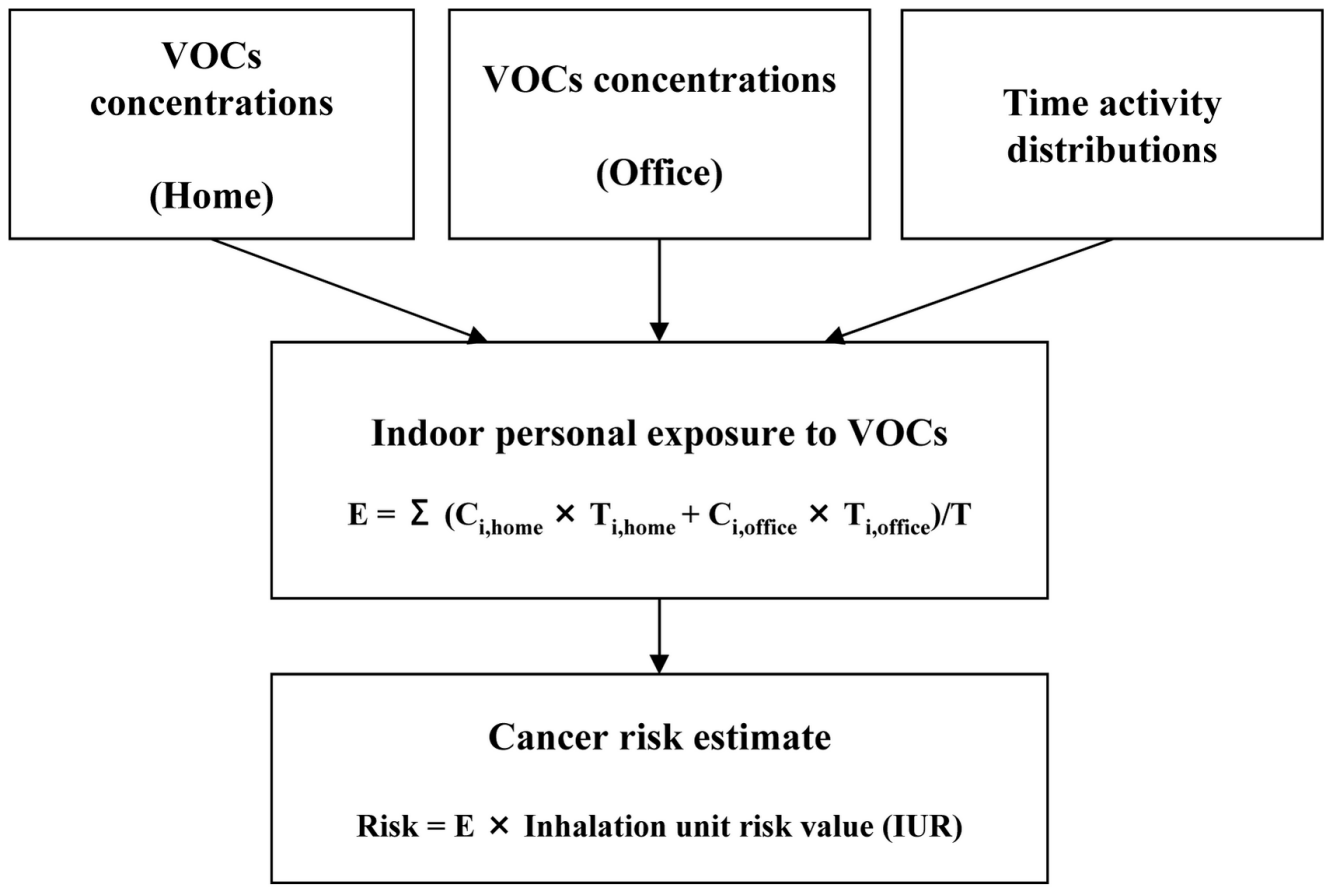
Diagram of personal exposure and cancer risk assessment model.

The model population in this study was adult males and females who live and work in newly remodeled buildings. We a) developed the distributions of indoor personal concentrations of formaldehyde and benzene using Monte Carlo Simulations in Crystal Ball (5,000 trials); b) calculated the cancer risks associated with the developed exposure to formaldehyde and benzene; c) compared the cancer risks to those reported in the literatures. Figure 2 illustrates the model framework. The indoor personal exposure to formaldehyde or benzene can be calculated using the following equation,
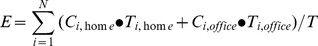
(2)where E is the indoor exposure to formaldehyde or benzene; C*_i,home_* is the concentration of formaldehyde or benzene for *i*th person at home; C*_i,office_* is the concentration of formaldehyde or benzene for *i*th person in office; T*_i,home_* is the time that *i*th person spends at home; T*_i,office_* is the time that *i*th person spends in office; T is total exposure time in all microenvironments, i.e. indoor and outdoor.

Distributions of the time that adult working males and females spend in home and office were taken from the *Time Use Patterns in China* [3]. Distributions of formaldehyde and benzene concentration were obtained using the concentration data in this study. We calculated risks by multiplying personal concentration by Inhalation Unit Cancer Risk values (IURs), which represent the excess number of cases per million people expected to develop cancer following lifetime (70 years) exposure to 1 µg/m^3^ of a given agent [10], [32]. Two sets of factor values were used given uncertainty in the toxicity estimates. One set is from the IRIS database (U.S. EPA 2010), and the values for formaldehyde and benzene in this system are 1.30×10^−5^ and 7.8×10^−6^ per million, respectively [21], [32]. It is necessary to note that the U.S. EPA IRIS system provides two IURs for benzene. The other is 2.2×10^−6^ per million [21], [32]. The higher IUR for benzene was used in our study to obtain a maximum estimate of cancer risk from benzene exposure. The other set is from the California OEHHA (California Environmental Protection Agency 2005), and the values for formaldehyde and benzene are 6.00×10^−6^ and 2.90×10^−5^ per million, respectively [21]. We disaggregated risks into home indoor and office indoor exposure parts.

## Results and Discussion

### Indoor Formaldehyde and Benzene Concentrations

**Figure 3:**
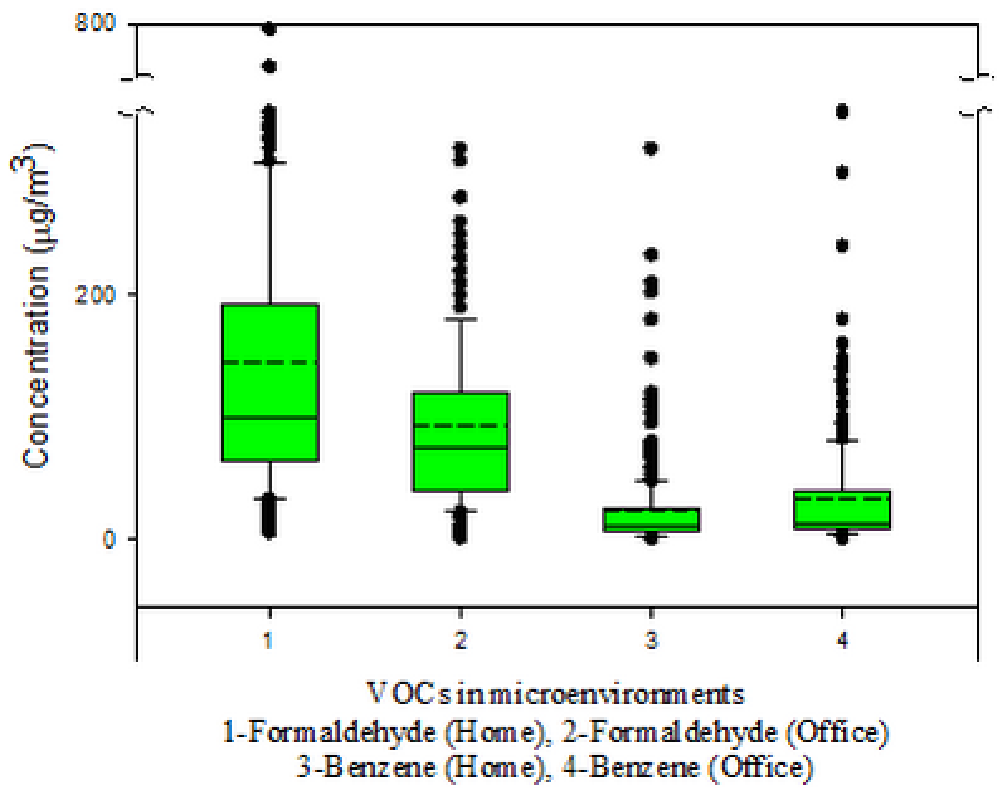
The concentrations of indoor formaldehyde and benzene in newly remodeled buildings in Beijing.

The concentrations of indoor formaldehyde and benzene are illustrated in Figure 3. The concentrations of formaldehyde were 131±90 (100) µg/m^3^ (Mean ± SD (Median), N = 383) in the tested dwellings and 85±56 (74) µg/m^3^ (N = 406) in the tested offices. The benzene data is more highly skewed than the formaldehyde data. Benzene concentrations were 17±16 (11) µg/m^3^ (Mean ± SD (Median), N = 379) in the tested dwellings and 30±34 (16) µg/m^3^ (N = 375) in the tested offices. Lognormal distribution is the best model that fits the formaldehyde and benzene data in our study. This is consistent with conventional concept on probability distribution of pollutant concentrations, which has been illustrated by Beaker Pouring Experiment [34]. The formaldehyde and benzene concentrations in this study are higher than the limited literature data for ordinary buildings (not recently remodeled) in China. Wang et al. [25] tested 3 homes in Beijing and found that the formaldehyde concentration ranged from 30 to 90 µg/m^3^. Jiang and Zhang (2012) measured indoor concentrations of carbonyls in 22 offices of the academic buildings, and the formaldehyde concentrations were 22.6±11.0 µg/m^3^ [35]. Zhou et al. [36] investigated the residential indoor benzene concentration in Tianjin, a mega-city next to Beijing, and the values were 6.13±7.58 (N = 10) in home environment and 1.38±0.57 (N = 6) in office environment. The comparison indicated that emission of benzene, formaldehyde and potential other VOCs from renovation and decoration materials led to the poor IAQ in the offices and dwelling investigated in the study.

The indoor concentrations of formaldehyde in our study were approximately two orders of magnitude higher than the outdoor formaldehyde concentrations in urban areas of Beijing, i.e. 7.8±3.8, 8.8±4.7 and 10.2±4.2 µg/m^3^ in 2008, 2009 and 2010, respectively [27]. This comparison indicated that formaldehyde emission from the indoor sources of newly remodeled buildings was much stronger than from outdoor sources. Given that urban residents in China spend more than 85% time indoor [3], exposure to indoor formaldehyde is expected to dominate personal formaldehyde exposure for urban residents who live and work in newly remodeled buildings. We also compared the indoor concentrations of formaldehyde by the two building types tested in the study. The concentrations of formaldehyde in the dwellings were significantly (p<0.01) higher than in the offices. This difference is due to the complex and intensive decoration and refurbishment that are very popular in Chinese dwellings [37]. Compared to relatively simple decoration in offices, the decoration in dwellings may result in greater formaldehyde emission from the decoration materials in the tested remodeled homes. It is interesting to find that the formaldehyde concentrations in newly remodeled dwellings in Beijing are similar to the formaldehyde concentrations in new homes in Korea and Japan, 134 µg/m^3^ (mean, based on the measurements from 292 new homes) [38]. In Europe, the formaldehyde concentrations measured in newly remodeled homes are generally lower, e.g. 40 µg/m^3^ (median) based on 367 measurements in newly prefabricated houses between 1996 and 2006 in Germany, and 37 µg/m^3^ (median) in 36 newly remodeled Danish apartments [38].

Benzene is widely used as solvents and adhesives [1], [13], [15]. The use of benzene in those products were banned or restricted in the developed countries when its carcinogenic effects were confirmed [15]. Thus, outdoor sources, such as vehicle emission and gasoline station [1], [20], generally make dominant contribution to benzene in indoor environment in the developed countries [10], [20], [23]. In our study, the indoor benzene concentrations, however, were noticeably higher than the ambient benzene concentrations in Beijing, 6.9±6.7 and 9.2±7.6 µg/m^3^ in 2008 and 2009 [26]. This comparison indicated that benzene emission from indoor sources of newly remodeled buildings in Beijing, same as formaldehyde, was much higher than outdoor sources. It is thus suspected that some adhesives and solvents containing benzene were continued used in decoration and renovation. Contrary to formaldehyde, benzene concentrations in the tested offices were significantly (p<0.01) higher than in the tested dwellings. This difference suggests that the adhesives and solvents containing benzene may be more frequently used in office decoration and renovation than in home renovation.

### Non-carcinogenic Health Risk Assessment

We compared the indoor concentrations of formaldehyde and benzene in the tested buildings to the health guidelines of China and USA. We note that the measured concentrations of the two compounds are for a 45-min period. As the concentrations were measured when the windows were closed and the impacts of human activities were minimized for 12–24 hours, the indoor concentrations of VOCs were expected to be stable, and thus our measurements were likely to be representative for the concentrations resulted from emission from decoration, renovation and refurbishment materials.

The RfCs of the *Chinese National Indoor Air Quality Standard* (GB/T 18883-2002) are 0.10 and 0.11 mg/m^3^ for formaldehyde and benzene, respectively. Formaldehyde concentrations in the tested buildings were above the RfC of GB/T 18883-2002 in 53% dwellings and 28% offices; whereas benzene concentrations were above the RfC of GB/T 18883-2002 in 2% offices and no dwellings had benzene concentration exceeding the guideline. The acute inhalation RELs of OEHHA were 55 and 1,300 µg/m^3^ for formaldehyde and benzene, respectively. The levels of benzene in all of the dwellings and offices met the guidelines, while 85% dwellings and 67% offices had indoor formaldehyde concentrations exceeding the guidelines. The chronic non-carcinogenic RELs of OEHHA were 9 and 60 µg/m^3^ for formaldehyde and benzene, respectively. The formaldehyde concentrations of all buildings were above the chronic RELs of OEHHA, whereas only 9.6% offices and no dwellings had benzene concentrations above the chronic RELs. The U.S. EPA IRIS system does not have chronic RfC for formaldehyde but benzene (30 µg/m^3^). The benzene concentrations of 12% dwellings and 32% offices exceeded the chronic RfC (U.S. EPA IRIS) for benzene.

The comparison to health guidelines highlights concern on the IAQ of these newly remodeled buildings that were investigated in our study. The exposure levels of formaldehyde in most tested indoor environments were very high, especially the dwellings. According to GB/T 18883-2002, ∼50% dwellings and ∼30% offices had formaldehyde concentrations above the standard. Based on the acute REL of OEHHA, the formaldehyde exposure in ∼80% dwellings and ∼65% offices may trigger acute adverse health effects. These acute effects include, but not limited to, eye, throat, and respiratory irritation, tearing, sneezing, coughing, chest congestion, fever, heartburn, lethargy, loss of appetite, and even asthma attacks [1], [12]. The scenario of chronic non-carcinogenic effects associated with formaldehyde exposure could be even worse. Based on the chronic REL of OEHHA, formaldehyde exposure in all tested dwellings and offices may result in chronic adverse health effects on the occupants. The chronic effects include headaches, dizziness, sleep disorders, memory loss, pulmonary function damage, pancytopenia and possible menstrual disorders of adult females [12]. The formaldehyde concentration could decrease and maintain stable at ∼35% of the initial concentration 3 years after remodeling [39]; however, 99% dwellings and 95% offices may still have formaldehyde concentrations above the chronic REL recommended by OEHHA even with a ∼65% decrease of exposure level with prolonged time.

The scenario of benzene is less severe than formaldehyde: only 2% offices did not meet the guideline set by GB/T 18883-2002. In terms of the stricter U.S. EPA IRIS guidelines, the benzene concentrations of all dwellings and offices met the guidelines of both OEHHA and U.S. EPA IRIS for acute adverse health risks while occupants have risks to develop chronic diseases in ∼10% dwellings and ∼30% offices. Therefore, the risks of developing chronic diseases are the major concern for benzene exposure in the tested dwellings and offices. The critical chronic effects of benzene exposure include decreased lymphocyte count, hematotoxicity and immunotoxicity (U.S. EPA IRIS).

### Cancer Risk Assessment

In our study, the indoor formaldehyde concentrations of all dwellings and offices exceeded the inhalation risk level corresponding to cancer risk of 100 excess cases per million (8 µg/m^3^, U.S. EPA IRIS). The indoor benzene concentrations of all dwellings and offices were above the inhalation risk level corresponding to cancer risk of 10 excess cases per million (1.3 µg/m^3^, U.S. EPA IRIS), whereas 48% dwellings and 69% offices had indoor benzene concentrations above the inhalation risk level of 100 excess cases per million (13 µg/m^3^, U.S. EPA IRIS). The comparison results indicated the potential cancer risks from exposure to formaldehyde and benzene. The cancer risks were quantitatively assessed based on modeled indoor personal exposure to the two compounds, which are presented below.

#### Indoor personal exposure to formaldehyde and benzene

**Figure 4:**
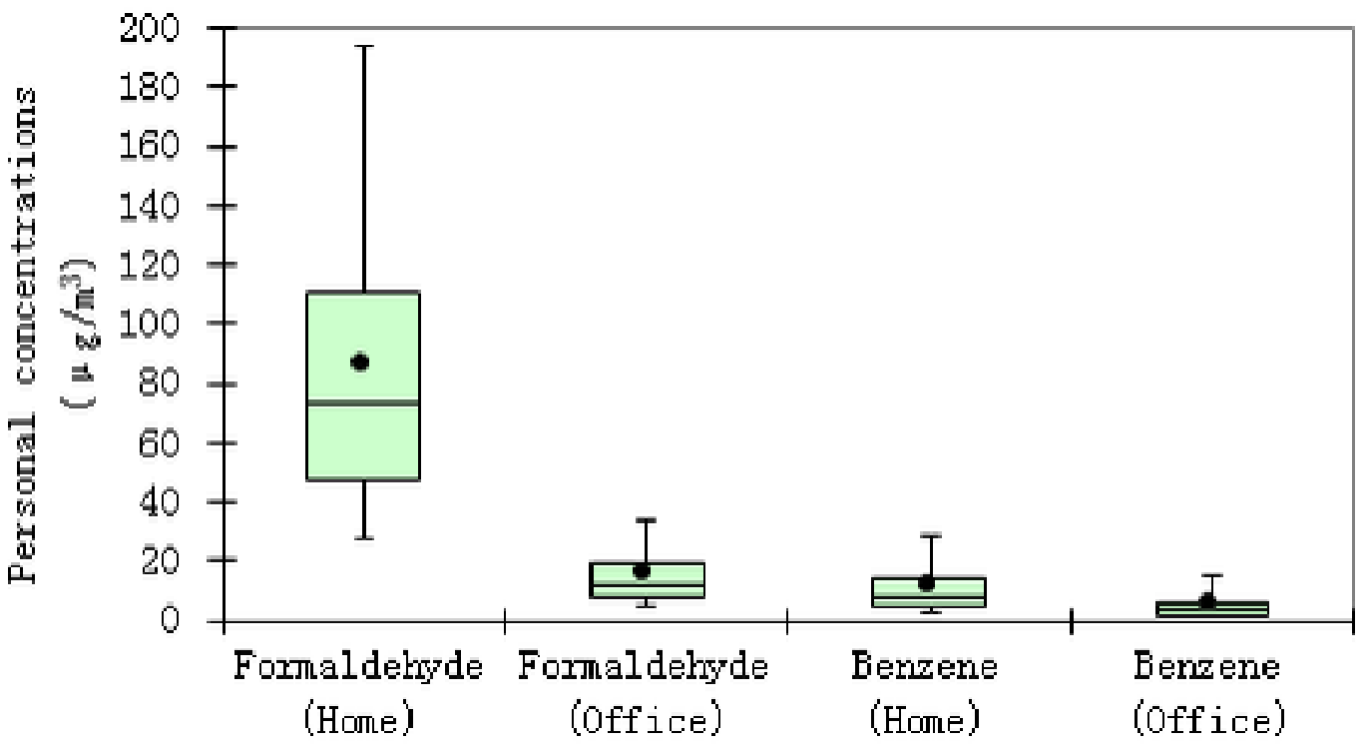
Personal concentrations of formaldehyde and benzene in dwellings and offices.

The indoor personal exposure to formaldehyde and benzene were modeled using Monte Carlo Simulation in Crystal Ball. The exposure distributions are illustrated in the Supporting Information (Figures S1–S4). We note that the modeled personal concentrations of formaldehyde and benzene were the indoor (home+office) fraction of total personal exposure for each of the two compounds. As shown in Figure 4, the personal exposure of formaldehyde in dwellings and offices were 86±52 (73) µg/m^3^ (Mean ± SD (Median)) and 15±9.1 (12) µg/m^3^, respectively; while the personal concentrations of benzene in dwellings and offices were 11±8.6 (8.3) µg/m^3^ and 5.0±4.7 (3.5) µg/m^3^, respectively.

#### Cancer risk assessment

We calculated the cancer risks from inhalation exposure to formaldehyde and benzene in indoor environment using both U.S. EPA IRIS and OEHHA IURs. The descriptive statistical information of the cancer risk estimates is summarized in Table 1. Based on the OEHHA IURs, the median cancer risks were 531 and 394 excess cases per million for formaldehyde and benzene, respectively, if model individuals live and work in newly remodeled homes and offices over lifetime. Based on the U.S. EPA IRIS IURs, the median cancer risks from indoor exposure to formaldehyde and benzene are 1,150 and 106 excess cases per million, respectively. We disaggregated the cumulative risks into the risk attributed to exposure at home and the risk attributed to exposure in office. The residential microenvironment accounts for ∼85% and ∼70% of the cumulative risks from indoor exposure to formaldehyde and benzene, respectively.

**Table 1:** The estimated cancer risks from indoor exposure to formaldehyde and benzene.

	City and country	Source of IURs^a^	Compound	Cancer risk (excess cases per 1 million population)			
				Mean	SD	Median	95%
Our study	Beijing, China	OEHHA^b^	Formaldehyde	604	314	531	1,260
			Benzene	463	285	394	1,030
		U.S. EPA IRIS^c^	Formaldehyde	1,340	771	1,160	2,880
			Benzene	131	96	107	304
Loh et al. [21] d	USA	OEHHA	Formaldehyde	100^e^			
			Benzene	108^e^			
		U.S. EPA IRIS	Formaldehyde	240^e^			
			Benzene	30^e^			
Sax et al. [20] d	New York City and Los Angeles, USA	U.S. EPA IRIS	Formaldehyde	205 (NYC), 258 (LA)^e^			
			Benzene	25 (NYC), 34 (LA)^e^			
Zhou et al. [36] f	Tianjing, China	U.S. EPA IRIS	Benzene	∼22^e^			

a IURs refers to Inhalation Unit Risk Values.

b OEHHA: California Office of Environmental Health Hazard Assessment.

c U.S. EPA IRIS: U.S. EPA Integrated Risk Information System.

d The cancer risk estimates from Loh et al. [21] and Sax et al. [20] represented the cancer risks associated with baseline exposure to formaldehyde and benzene in both indoor and outdoor microenvironments.

e Median cancer risk values are provided for Loh et al. [21], Sax et al. [20] and Zhou et al. [36].

f The cancer risk estimates from Zhou et al. [36] represented the cancer risks associated with exposure to benzene in both indoor and outdoor microenvironment.

The calculated cancer risks, to our knowledge, are the highest reported in scientific literature. The comparison is shown in Table 1. With regards to the cancer risks from inhalation exposure to formaldehyde and benzene, our values were ∼5 times and ∼3 times of Loh et al. [21] (Table 1). It is necessary to point out that the values yielded by the model calculation in Loh et al. [21] represent the cancer risks from baseline exposure in both indoor and outdoor microenvironments. Sax et al. [20] assessed the cancer risks from inhalation exposure to VOCs for non-smoking teenagers from non-smoking homes in New York City and Los Angeles. The assessment was based on the measured personal concentrations of VOCs. The risks in Sax et al. [20] were about one fourth (formaldehyde) and one third (benzene) of the values in our study (Table 1). Again, the risks in Sax et al. [20] also represent the cancer risks from exposure in both indoor and outdoor environments. Zhou et al. [36] assessed the cancer risks based on the measured personal concentrations of BETX for 12 adults in Tianjin, China. The cancer risk (all microenvironments, indoor+outdoor) attributed to benzene in their study was lower than our value as well (Table 1). Note: only 5 participants in Tianjin Study renovated their apartments within the past year [36]. In addition, indoor concentrations of formaldehyde and benzene in the newly remodeled properties in our study were much higher than outdoor counterparts as previously discussed. Thus, it is not surprising that the cancer risks of indoor exposure were much greater than the values that were reported for outdoor exposure only in Beijing, i.e. 91.1 (formaldehyde) and 41.9 (benzene) per million [26].

It is necessary to note again that the concentrations of indoor formaldehyde and benzene will decrease with time after remodeling. Therefore, the risk values that we obtained through model calculation represent the cancer risks for the highest exposure scenario. Nonetheless, Ohura et al. [23] found that indoor benzene concentration in China dropped to ∼25% of initial concentration and maintained stable 3 months to 1 year after remodeling, and Zhao et al. [39] suggested that indoor formaldehyde concentration in China dropped to ∼35% of the initial concentration and maintained stable 3 years after remodeling. Assuming formaldehyde and benzene concentrations in the tested buildings in our study decreased to ∼35% and ∼25% of the reported values, respectively, the cancer risks associated with indoor exposure to formaldehyde and benzene would decrease to ∼406 and ∼27 excess cases per million (based on U.S. EPA IRIS 2010), respectively. These values, however, are still noticeably higher than those reported by researchers in the U.S. (Table 1). Our study results showed that cancer risks associated with baseline inhalation exposure to indoor formaldehyde and benzene in these Chinese buildings in our study may be significantly higher than the cancer risks associated with the baseline formaldehyde and benzene exposure in the U.S.

### Uncertainties and Limitations

Our study assessed the health risks from indoor formaldehyde and benzene exposure in recently remodeled dwellings and offices in Beijing. Occupants of these buildings requested for the IAQ tests due to a variety of reasons, which included, but not limited to, uncomfortable odor, awareness of potential unhealthy IAQ in newly remodeled buildings and its impact on health. Therefore, the assessment results reflect the scenario of the newly remodeled buildings in Beijing, although the measurements were obtained from those requested for IAQ test.

Since the dwelling windows were closed 12–24 hours prior to field tests until completion, the ventilation rates were very low during the tests. As previously discussed, such an airtight state is prevalent in the winter and summer for Beijing residential spaces. This is because windows are usually closed when either central heating or air conditioning is on. Unlike the two seasons, Beijing residents frequently open windows for ventilation in the spring and fall. As a result, indoor exposure levels of formaldehyde and benzene and the associated health risks in newly remodeled dwellings may be lower in these two seasons. Nonetheless, using the summer and winter data can yield maximum estimate of health risks. Residents would actually benefit from regulation developed based on the maximum health risk estimate.

## Conclusions and Recommendations

This study reports the formaldehyde and benzene levels in newly remodeled dwellings and offices in Beijing, in which the occupants have health concerns about IAQ. The concentration data and subsequent health risk assessment can help us understand the health risks associated with the IAQ of these buildings. Exposure to formaldehyde may pose both acute and chronic non-cancer risks to the occupants in the tested buildings, as 85% dwellings and 67% offices had concentrations over the acute REL of OEHHA and all dwellings and offices over the chronic REL of OEHHA. Exposure to benzene may pose chronic non-cancer risks to the occupants, as 12% dwellings and 32% offices had concentrations over the chronic RfC of US EPA IRIS. The median cancer risks (per million) of indoor exposure to formaldehyde and benzene were estimated to be 1,150 and 106 (based on US EPA IRIS IURs), 531 and 394 (based on OEHHA IURs) if adult males and females work and live in the newly remodeled indoor environment over lifetime. Based on our assessment results, inhalation exposure to VOCs in newly remodeled buildings, which were mainly emitted from decoration and renovation materials, may trigger significant adverse health effects on occupants in China. Ventilation improvement is one of the potential strategies that can be considered to reduce indoor exposure to VOCs, especially for the airtight dwellings in Beijing. Another strategy would be the reduction of VOCs emission from building materials, decorating materials and furniture. For instance, the establishment of *Chinese indoor decorating and refurbishment materials and furniture VOCs emission labeling system* is recommended. This labeling system can serve as guidance for the consumers on selection of building and decoration materials.

There are still large knowledge gaps in the associations between indoor VOCs exposure and public health. For instance, current *Chinese National Indoor Air Quality Standard* (GB/T 18883-2002) does not involve some important carcinogenic VOCs species such as 1,4-dichlorobenzene, chloroform, 1,3-butadiene, acetaldehyde and tetrachloroethylene. Therefore, we recommend measuring more VOCs species in prospective IAQ monitoring campaigns and assessing associated health risks in China. We also recommend assessing the cancer risks from exposure to VOCs across various microenvironments and across various VOC species. Results of these research activities will provide a full scenario of Chinese environmental health that is associated with indoor VOCs pollution. These results will facilitate prioritization of air toxics for environmental regulation and pollution control, and ultimately protect public health in China.

## Supporting Information

Figure S1 **Distribution of personal exposure to formaldehyde in dwellings.**
(TIF)

Figure S2 **Distribution of personal exposure to formaldehyde in offices.**
(TIF)

Figure S3 **Distribution of personal exposure to benzene in dwellings.**
(TIF)

Figure S4 **Distribution of personal exposure to benzene in offices.**
(TIF)

Table S1 **Relevant guidelines and standards for indoor formaldehyde and benzene.**
(DOCX)
