# Supplementary figures and images for: Health Risk Assessment of Inhalation Exposure to Formaldehyde and Benzene in Newly Remodeled Buildings, Beijing
Source: PLoS One. 2013 Nov 14;8(11):e79553. doi: 10.1371/journal.pone.0079553 (PMC3828412; doi:10.1371/journal.pone.0079553)

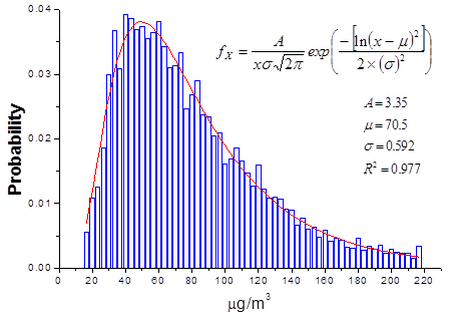

Supplement: Figure S1 — Distribution of personal exposure to formaldehyde in dwellings. (TIF) [file pone.0079553.s001.tif]

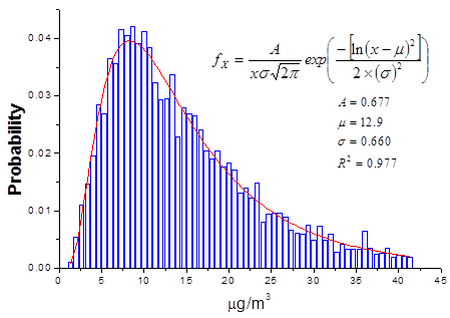

Supplement: Figure S2 — Distribution of personal exposure to formaldehyde in offices. (TIF) [file pone.0079553.s002.tif]

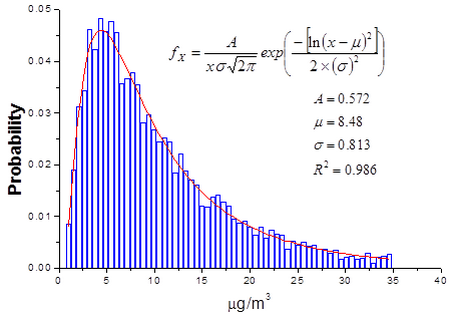

Supplement: Figure S3 — Distribution of personal exposure to benzene in dwellings. (TIF) [file pone.0079553.s003.tif]

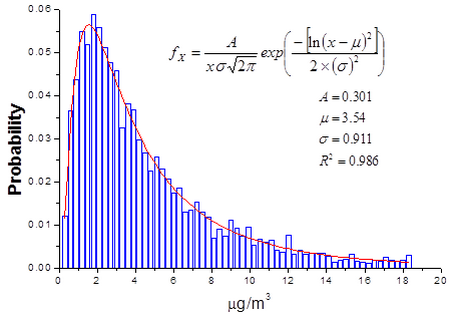

Supplement: Figure S4 — Distribution of personal exposure to benzene in offices. (TIF) [file pone.0079553.s004.tif]
