# Supplementary material for: Health Risk Assessment of Inhalation Exposure to Formaldehyde and Benzene in Newly Remodeled Buildings, Beijing
Source: PLoS One. 2013 Nov 14;8(11):e79553. doi: 10.1371/journal.pone.0079553 (PMC3828412; doi:10.1371/journal.pone.0079553)
Supplement: Table S1 — Relevant guidelines and standards for indoor formaldehyde and benzene. (DOCX) [file pone.0079553.s005.docx]

Table S1. Relevant guidelines and standards for indoor formaldehyde and benzene

| Source |  | Formaldehyde | Benzene |
| --- | --- | --- | --- |
| GB/T 18883-2002, China P.R. |  | 0.10 mg/m^3^ | 0.11 mg/m^3^ |
| U.S. EPA IRIS 2010 ^a^ | Non-carcinogenic RfC for chronic inhalation exposure | N/A | 30 µg/m^3^ |
|  | Carcinogenic Inhalation Risk Level for 1/1,000,000 | 0.08 µg/m^3^ | 0.13 µg/m^3^ |
|  | Carcinogenic Inhalation Risk Level for 1/100,000 | 0.8 µg/m^3^ | 1.3 µg/m^3^ |
|  | Carcinogenic Inhalation Risk Level for 1/10,000 | 8 µg/m^3^ | 13 µg/m^3^ |
| OEHHA 2008, USA | Acute Inhalation RELs ^b^ | 55 µg/m^3^ | 1,300 µg/m^3^ |
|  | Chronic Inhalation RELs ^c^ | 9 µg/m^3^ | 60 µg/m^3^ |

1. Source: http://www.epa.gov/iris/
2. The average time for acute RELs is 1-hour intermittent exposure

Chronic RELs are intended to address continual exposure over a lifetime which is measured by annual average exposure
